# Effect of ENSO events on larval and juvenile duration and transport of Japanese eel (*Anguilla japonica*)

**DOI:** 10.1371/journal.pone.0195544

**Published:** 2018-04-10

**Authors:** Kuan-Mei Hsiung, Shingo Kimura, Yu-San Han, Aigo Takeshige, Yoshiyuki Iizuka

**Affiliations:** 1 Graduate School of Frontier Sciences/Atmosphere and Ocean Research Institute, The University of Tokyo, Chiba, Japan; 2 Institute of Fisheries Science and Department of Life Science, College of Life Science, National Taiwan University, Taipei, Taiwan; 3 National Research Institute of Far Seas Fisheries, Fisheries Research Center, Kanagawa, Japan; 4 Institute of Earth Sciences, Academia Sinica, Taipei, Taiwan; Institute of Marine Research, NORWAY

## Abstract

Spawning ground of Japanese eel (*Anguilla japonica*) is located near the West Mariana Ridge seamount. The species travels through the North Equatorial Current (NEC) and then enters the Kuroshio Current (KC) on the migration toward East Asian growth habitats. Therefore, El Niño–Southern Oscillation (ENSO) events serve as the potentially important drivers of interannual variability across the equatorial Pacific. Because the NEC bifurcation and salinity profiles are related to ENSO events, we investigated the influence of locations of the NEC bifurcation and salinity front on the success of larval entry to the KC by numerically modeling particle transport in ocean currents from 1972 to 2013 and possible effects on the size of glass eels at continental recruitment and, via otolithometry on the duration of larval migration. Circulation and hydrography used for particle tracking were obtained from the results of the Model for Interdisciplinary Research on Climate (MIROC) high-resolution forecasting experiment. Our results demonstrated that during El Niño years, (1) the southward movement of the salinity front might cause the larvae to experience slower currents and (2) the northward movement of the NEC bifurcation might broaden the separation between their spawning ground and NEC bifurcation, thus prolonging the time needed for the larvae to enter the KC from their spawning ground, because of which the duration of entrainment in the water column and body size increase when eels reach estuarine waters. In addition, this might cause more water to flow into the Mindanao Current (MC), leading to a decline in the rate at which larvae get entrained into the KC.

## Introduction

**Fig 1:**
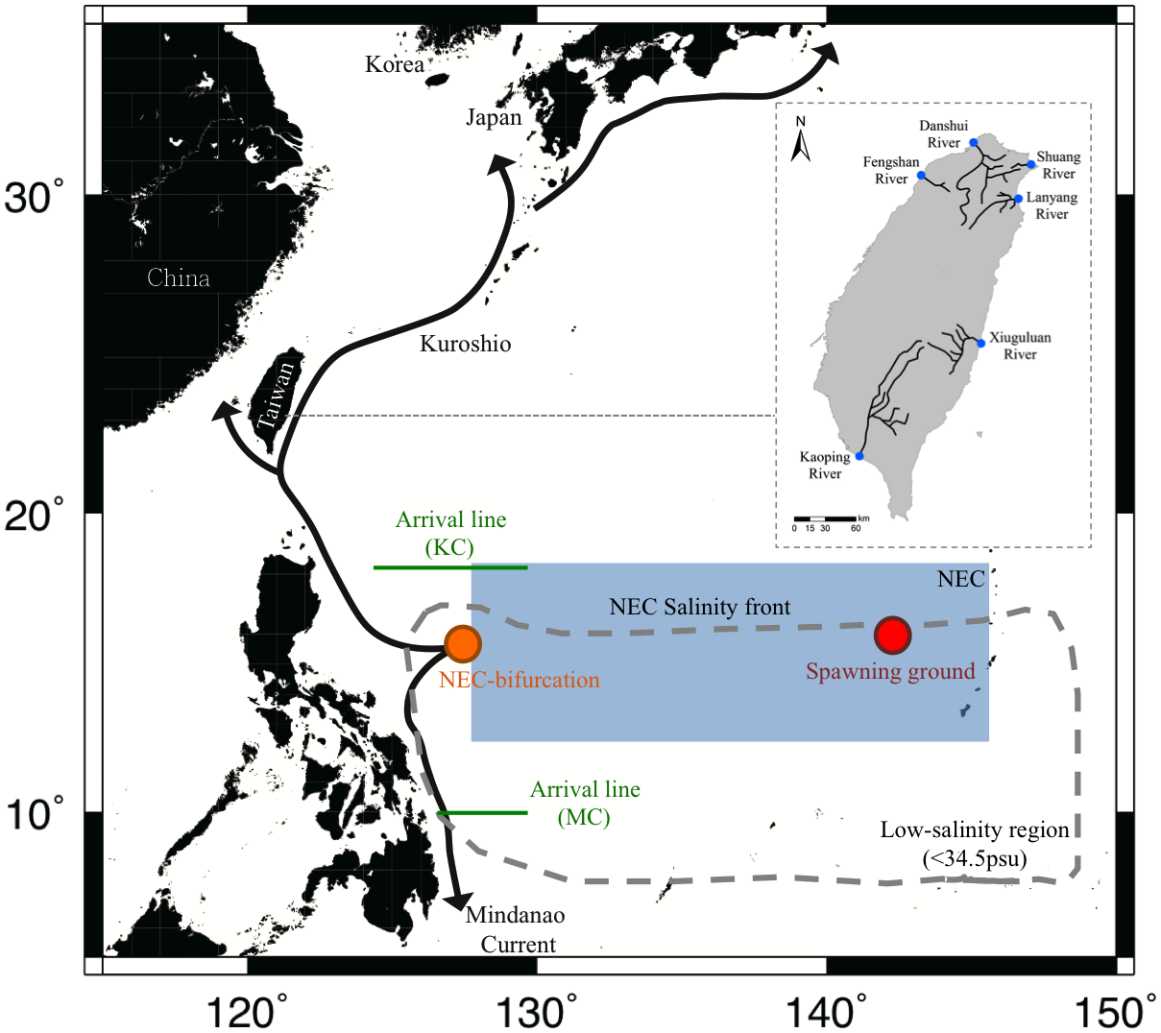
Schematic view of salinity structure, oceanic circulation and the North Equatorial Current (NEC) bifurcation in the western North Pacific Ocean related to the presumed spawning location of *A. japonica*. Blue spots in the inserted map show the catch sites of glass eel in Taiwan.

The Japanese eel, *Anguilla japonica* (Temminck & Schlegel), is mainly distributed in Taiwan, China, Korea, and Japan [1–3]. It is a commercially important freshwater fish in East Asia. However, all the eel fry needed for aquaculture depends on the catch of glass eels harvested in estuarine/coastal waters during their upstream migration. The spawning area of the Japanese eel was discovered near 14°-17° N, 142°-143° E in the North Equatorial Current (NEC) to the west of the Mariana Islands, approximately 3000 km away from their growth habitats in East Asia (Fig 1) [4, 5].

Mature eels mainly spawn between May and August [3, 4, 6]. After hatching, the larvae (leptocephali) passively drift from spawning site on the NEC to the Kuroshio Current (KC) for 4–6 months before reaching the East Asian coast [3, 6, 7]. Leptocephali are transported by currents, which are mainly 50–150m deep [8], and they metamorphose into glass eels as they approach continental growth habitats. Glass eels then show a benthic–sheltering behavior [1, 6, 8] and actively swim toward nearby estuaries and rivers for further growth [6].

Japanese eel recruitment has declined in most habitats since the late 1970s, with large inter-annual fluctuations [9–11] and is now listed as endangered on the International Union for Conservation of Nature Red List [12]. A combination of factors has been invoked to explain stock decline of *A. japonica*, including overfishing, environmental degradation of habitats [10, 13–15], and fluctuations in oceanic conditions [10, 11, 16–19].

Environmental variability affects the distribution, migration, and abundance of fish. The most obvious driver of interannual variability across the equatorial Pacific is the El Niño–Southern Oscillation (ENSO) event. Kimura et al. [16] found that Japanese glass eel catches in Japan have declined associated with El Niño events. Based on larval distribution patterns, it has been inferred that *A. japonica* spawns to the south of the NEC salinity front [16, 18](Fig 1), and that its larval transport in the NEC controls eel abundance in East Asian countries [18–20]. Therefore, changes in position of the salinity front have been suggested to be crucial to the spawning migration of Japanese eel [18].

The NEC bifurcates into the north-flowing KC and the south-flowing Mindanao Current (MC) at its westernmost boundary, off the coast of the Philippines [21, 22]. In the bifurcation zone, Japanese eel larvae need to enter the northward flow leading to the KC and avoid being entrained into the south-flowing MC where the environmental conditions are not favorable to the Japanese eel [19, 23]. More NEC water flows into the MC when the NEC bifurcation moves northward during an El Niño event, thus causing a decrease in the probability of eel larvae being transported into the KC; the opposite current pattern occurs during La Niña years [23].

Furthermore, Zenimoto et al. [24] demonstrated that annual catch per unit effort (CPUE) of *A. japonica* glass eels at Tanegashima Island which located in the southern part of Japan (30.3500° N, 130.5900° E) is lowest in an El Niño year and relatively high in a La Niña year. These observations indicate that shifts in the NEC bifurcation location might be related to ENSO events; such a relationship would further impact the duration of the larval stage and success rate of glass eel recruitment.

Research on otolith growth patterns of larvae and juvenile fish was improved by the discovery of daily growth increments [25]. Pannella [25] further investigated the otolith microstructure when he noted that CaCO_3_ is concentrically deposited in otoliths on a daily basis. Otolith ring pattern is now used to study daily, monthly, seasonal, and annual growth rates in a wide array of fish species from both freshwater and saltwater [25–27]. Because growth increments in otoliths of both newly hatched larvae and glass eels are deposited daily [28], the age of anguillid leptocephali and glass eels can be estimated by counting otolith increments. Hence, otolith microstructure analysis has emerged as a useful technique for revealing details about the early life history of anguillid eels [29].

According to previous studies, ENSO events affect the latitude of the salinity front [18, 30, 31] and location of NEC bifurcation [17]; hence, such events might impact the position of the eel spawning ground and velocity of the NEC and KC. Moreover, mean total length (TL) of *A. japonica* glass eels is longer during El Niño years and shorter during La Niña years [32].

This study aimed to elucidate whether ENSO events prolong the duration of eel transport and inhibit the subsequent recruitment of eel larvae from their spawning ground to the East Asian coast during El Niño years, and whether the converse occurs during La Niña years. This study analyzed the daily increments in otolith growth (sagittae) to elucidate the duration of the larval stage in Japanese eel. Then, the Lagrangian numerical simulation model of particle transport was used to investigate the interannual variability of the drifting time and the distribution patterns of larvae, thus estimating the proportion of larvae transported from the spawning area into the KC and MC. Finally, by comparing differences in the larval stage duration, body size, and recruitment success rate among El Niño, La Niña, and normal years, this study assessed possible impacts of ENSO events on larval migration in Japanese eel.

## Materials and methods

### Sample collection for total length analysis

A total of 10,768 *A. japonica* glass eel specimens were collected using a hand-trawling net for 2 h at night before high tide, 2–3 times monthly along the estuaries of the Lanyang River (24.7252° N, 121.8329° E), Danshui River (25.2182° N, 121.4372° E), Shuang River (25.0257° N, 121.9440° E), Fengshan River (24.9318° N, 120.9770 °E), Xiuguluan River (23.4660° N, 121.5022° E), and Kaoping River (22.4774° N, 120.4310°E) in Taiwan (Fig 1). Trawling was conducted during the fishing season (November–March) from 1984 to 2013. Commercial glass eel harvesting was undertaken at all the sampling sites. After collection, the captured glass eels were immediately preserved in 95% ethanol. The methods used to capture the glass eels were approved by the Fisheries Agency of Executive Yuan, Taiwan. Glass eels measurements. Measurements of *A. japonica* glass eels were performed after one month of storage when the samples stopped shrinking [33]. TL of specimens were measured to the nearest millimeter, and their pigmentation stages were observed based on techniques established by Tesch [1].

### Sample collection for otolithometry

According to previous studies, in Taiwan, the recruitment of Japanese glass eels usually begins in late October and ends in early April of the following year [34, 35]. Therefore, we randomly sampled about 20 individuals per year among nine annual recruitment events of glass eels between November and the following March for otolith increment analysis from six sites between 2002 to 2013 (Table 1). These events were separated into three groups: El Niño, La Niña, and normal climatic years, with each group containing three recruitment events. Due to the limited numbers of otoliths obtained between 2004 to 2006, we excluded the otoliths from this our dataset.

**Table 1:** The details of sampling time, sampling location, climate status, sample size, the mean total length (TL) and the mean larval duration (LD) obtained from otolith daily increments analysis of *A. japonica* glass eel in the nine ENSO climate periods. The mean TL was measured after 1+ months of preservation shrinkage.

Year	Climate	Sampling sites	Month, Year	N	Total length (mm) Mean ± SD	Larval duration (day) Mean ± SD
2002-2003	El Niño	Kaoping River, Taiwan	Jan., 2003	19	55.6 ± 2.1	143.3 ± 9.1
2003-2004	El Niño	Danshui River, Taiwan	Dec., 2004	20	57.2 ± 1.7	148.4 ± 8.4
2006-2007	Normal	Shuang River, Taiwan	Jan., 2007	20	55.5 ± 1.4	125.9 ± 5.3
2007-2008	La Niña	Fengshan, Taiwan	Nov., Dec., 2007	19	55.6 ± 1.6	126.9 ± 9.5
2008-2009	Normal	Kaoping River, Taiwan	Feb., 2009	19	56.2 ± 1.2	128.8 ± 2.8
2009-2010	El Niño	Danshui River, Taiwan	Mar., 2010	23	55.3 ± 0.8	146.7 ± 14.0
2010-2011	La Niña	Lanyang River, Taiwan	Dec., 2010	21	55.0 ± 1.8	124.8 ± 10.0
2011-2012	La Niña	Lanyang River, Taiwan	Nov., 2011	21	57.6 ± 0.6	131.2 ± 1.5
2012-2013	Normal	Lanyang River, Taiwan	Jan., 2013	20	56.1 ± 1.3	126.0 ± 5.6

### Otolith preparation

The sagittal otoliths were extracted from each individual and put into an embedding template. The template was then put into a vacuum machine and an oven for 3–5 days to remove moisture from the otoliths. After subjecting them to vacuum and drying again for 2 hours, they were embedded in an epoxy resin, mixed with epoxy harder in the proportion of 5:1, and mounted on glass slides using a glue gun. Embedded otoliths were ground nearly to the core using a grinding machine with 1200 grit (P2500) sandpaper. Otoliths were then polished using a cloth with a 0.05 and 1-*μ*m alumina slurry along the anterior–posterior direction of the frontal plane of fishes until the core was exposed and scratches on the otoliths were smoothed. Then, the otoliths were rinsed with distilled, deionized water for 10–15 s, and the surfaces of otoliths were carefully cleaned with lens paper soaked in 100% ethanol. After we confirmed that the surfaces of the otoliths were very clean and the cores could be seen clearly, they were etched with 0.05M HCl for 5 s. Finally, they were placed in an oven at 65°C for 3–5 days [5, 36]. During this period, the otolith surfaces were mopped with lens paper and 100% ethanol every day to remove any moisture remaining on the otoliths.

### Scanning electron microscope (SEM)

The otoliths were vacuum-coated with Au in an ion sputterer prior to observing them with SEM (JSM-6360LV, JEOL), which was equipped with an energy-dispersive X-ray spectrometer (EDS: Oxford EDS, Xmax 80), in the electron probe micro-analysis laboratory of the Institute of Earth Science, Academia Sinica. The images were photographed at a magnification of 300× and 2000× for observing otolith microstructure at each larval stage and for counting the number of daily growth increments.

### Otolith increment analysis

**Fig 2:**
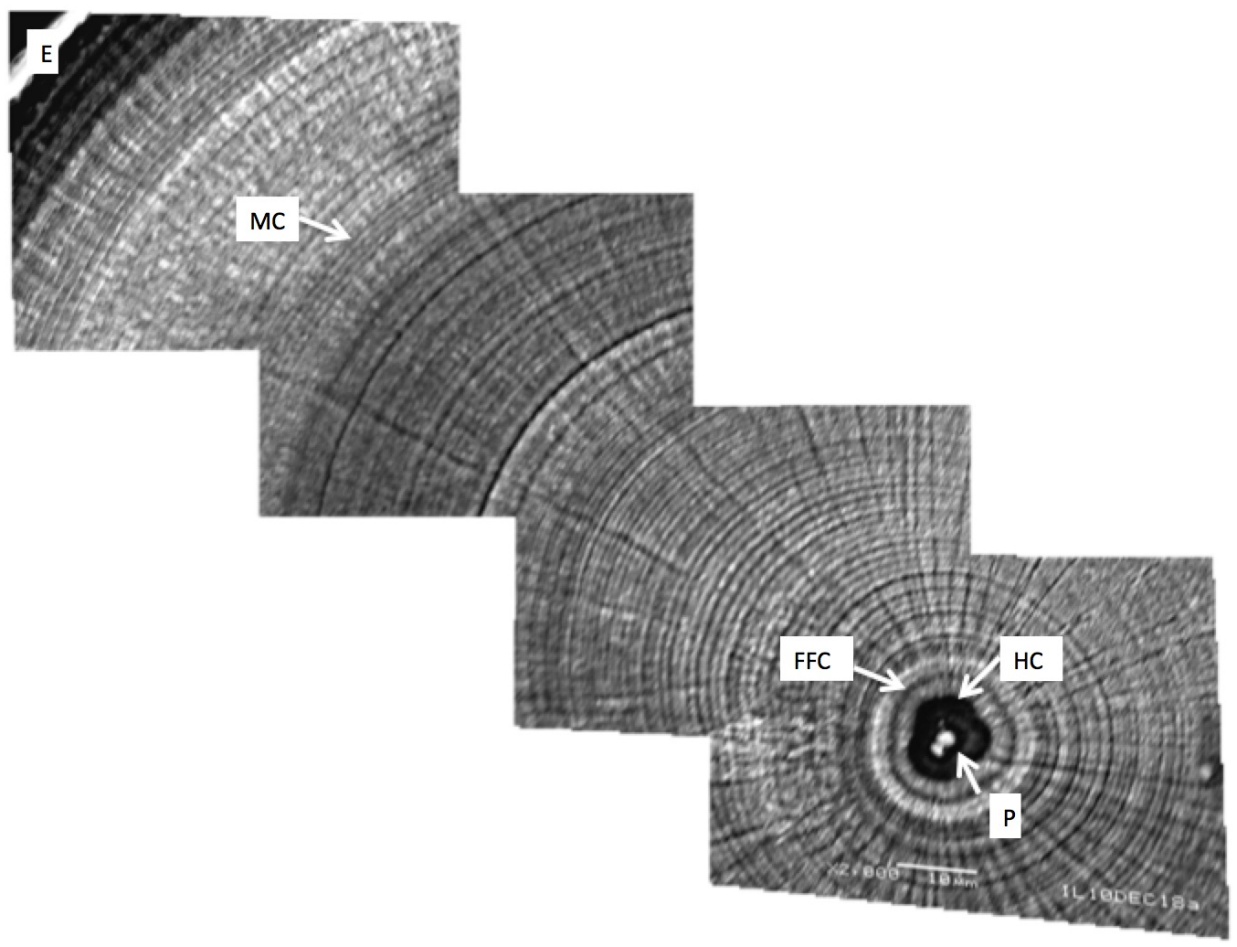
SEM photograph of the otolith of *A. japonica* glass eel. Scale bar = 10μm. P: primodium; HC: hatching check; FFC: first feeding check; MC: metamorphosis check; E: edge. The definition of the onset in each edge was modified from Arai et al. [66].

Otolith images were photographed at 300× magnification to observe the entire otolith and select the optimal portion for further investigation. Then, SEM photographs were recorded at 2000× magnification and combined to observe otolith microstructure and count the number of daily increments. The daily increments of *A. japonica* were counted from the primodium to the metamorphosis check mark (Fig 2).

### Data source of ENSO events

The El Niño/La Niña events were identified from the data collected by the Climate Prediction Center of the National Oceanic and Atmospheric Administration (NOAA, Washington DC, USA) (http://www.cpc.ncep.noaa.gov/products/analysismonitoring/ensostuff/ensoyears.shtml). Monthly values during the years 2003–2013 were based on the Oceanic Niño Index (ONI), which is derived from a 3-month running mean of ERSSTv4 sea surface temperature anomalies in the Niño 3.4 region located at 5° N–5° S, 120°–170° W. El Niño events were defined when a threshold temperature of +0.5°C for the ONI was met for a minimum of 5 consecutive overlapping months. La Niña events were defined when a threshold temperature of −0.5°C for the ONI was met for a minimum of 5 consecutive overlapping months.

### Circulation model

A Lagrangian study of *A. japonica* larval migration was conducted using results of the Model for Interdisciplinary Research on Climate (MIROC) high-resolution forecasting experiment [37, 38]. MIROC forecasting experiments were conducted under the IPCC A1B carbon dioxide emission scenario of the Special Report on Emission Scenarios [39]. The A1B scenario describes a future undergoing rapid economic growth, with the global population peaking at mid-century, declining thereafter, and affected by the rapid introduction of new and more efficient technologies.

MIROC version 4h was used in this study, which is a new atmosphere/ocean-coupled general circulation model (AOGCM) developed by the Atmosphere and Ocean Research Institute (AORI) of the University of Tokyo, National Institute for Environmental Studies (NIES), and Japan Agency for Marine-Earth Science and Technology (JAMSTEC) [40]. The Climate System Research (CCSR) Ocean Component Model (COCO, version 3.4) was used to provide the ocean component of MIROC 4h, a detailed description of which can be found in Hasumi [41]. Model topography was generated from a widely used 2-minute resolution bathymetry data set (ETOPO2). MIROC 4h consists of a 0.1° horizontal grid and 47 vertical layers, the upper eight layers of which are within the *σ*-coordinates. The vertical grid spacing varies with depth [40]. It contains data of water temperature, salinity, and horizontal and vertical current velocities.

In a historical experiment, a set of the 20th century (20C3M) model to simulate climate changes during the 20th century and assess how well the model reproduced past changes in climate was used in MIROC 4h. This model provided the initial conditions for our scenario experiments. For MIROC 4h, a control experiment was conducted using fixed, external forcings at levels prevalent during the 1950, and 20C3M was run by changing the external forcing from 1950 to 2005 [42]. The time-step of the model integration was 3 s for the barotropic mode and 3 min for other modes.

### Particle tracking

A particle tracking experiment was conducted to determine differences in larval transport between ENSO and normal climatic years. A three-dimensional (3D) advection–diffusion scheme was used. The position of a particle was tracked from its position at time “*t*” to a new position at time “*t* + *δt*” based on the following equation:

$$\begin{array}{c} X_{p}\left(t+\delta t\right)=\left[X_{p}\left(t\right)+u\left(t\right)\delta t\right]+\delta l_{diff}, \end{array}$$ (1)

To calculate the advection of particles, velocity *u* (*x*_*p*_, *y*_*p*_, and *z*_*p*_) was used, where *z*_*p*_ = 50 and 150 m, which was weighted by the distances from each grid point for four velocities in each grid field. *deltal*_*diff*_ represents additional displacement during a time interval (*t*) associated with a random walk. For diffusion of particles, 100 *m*^2^*s*^−1^ was adopted as the horizontal eddy diffusivity [24].

Previous studies showed that the spawning season of the Japanese eel is mainly from May to August [36, 43–45]; thus, 10000 particles were released at model dates June 1, July 1, and August 1 and then tracked for 360 days.

Kimura et al. [16] indicated that the spawning site of this species might be located on the southern side of the salinity front, indicating that the spawning location varies according to the position of the salinity front. Because most small larvae of *A. japonica* were collected between 13° N and 15° N [4, 7, 29], we modelled two start locations in our numerical simulations: (1) fixed at 14° N and (2) at the latitude of the NEC salinity front along the 142° E longitude. The transport process was simulated from 1972 to 2013 using the velocity fields of each year.

Diel vertical migration, which is an important biological behaviour of the Japanese eel, was also considered in this study. According to previous studies, eel larvae remain in upper surface waters at night (approximately 50-m deep) and evade predators during daytime by diving into deeper waters (approximately 150-m deep) [46–48]. Therefore, the vertical distribution in this study was fixed at a depth of 150 m during the day and 50 m during the night.

In our research, particles entering into the KC(18° N, 122–127° E) were counted as individuals successfully entrained into the KC, whereas particles entering into the Midanao Current (10° N, 125–130° E) were counted as recruitment failures.

### Statistical analysis

Differences in parameters, such as mean TL, mean larval duration (LD), particle transport success rate (%), and mean drifting time (day) were evaluated. These parameters included comparisons of each climate category and model year using the one-way analysis of variance (ANOVA), followed by Tukey’s honest significant difference (HSD) multiple comparison test. Statistical Package for the Social Sciences (SPSS) 16.0 software was used for statistical analyses. Differences were considered significant when *p* was <0.05. In addition, the long-term annual variation of TL and LD was tested using regression analysis. Furthermore, to determine if our results of particle tracking corresponded to the observed data, we compared the results of particle tracking and Japanese glass eel glass eel catch data of East Asia from 1972 to 2013 reported by the Nihon Yoshoku Shimbun (Japan Aquaculture newspaper) which could be obtained by paid subscription or fax services for readers overseas.

## Results

### Relationship between mean TL and mean LD

**Fig 3:**
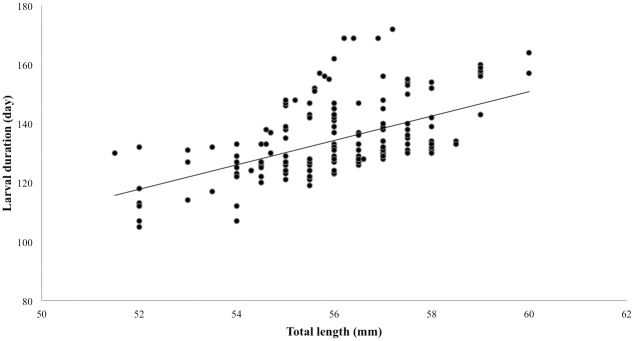
The regression analysis of the relationship between the mean larval duration and the mean total length (N = 182, r = 0.40, *p* < 0.05).

The *A. japonica* specimens used to measure the LD and TL in this study were collected from the estuaries of Taiwan over a 9-year period (Table 1). A regression analysis was applied to understand the relationship between TL and LD. The results revealed that the relationship between mean TL and mean LD showed statistically positive correlation (r = 0.40, *p* < 0.05) (Fig 3).

### The influence of El Niño and La Niña events on the LD of glass eel

**Fig 4:**
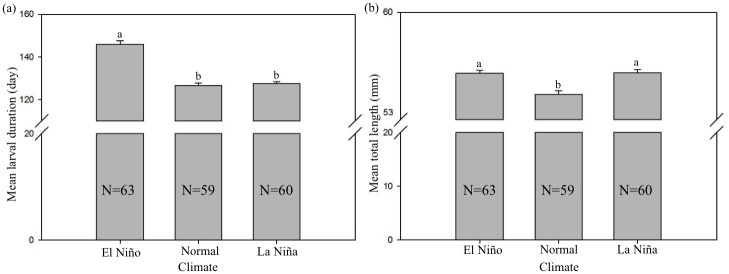
Comparison of the (a) mean larval duration (day) and (b) mean total length (mm) of *A. japonica* glass eels among the nine selected years in Taiwan. Numbers within bars indicated sample size. Bar charts with different letters above are significantly different (Duncan, *p* < 0.05) between different ENSO climate periods.

To a certain extent, LD oscillation appears to be related to ENSO events; the mean LD in El Niño years (146.2 ± 11.0 days) was significantly higher than that in normal (126.8 ± 4.9 days) and La Niña (127.6 ± 8.3 days) years (Duncan, *p* < 0.05; Fig 4a). No significant difference was recorded between LDs obtained in normal and La Niña years. In contrast, the mean TLs in El Niño and La Niña years were significantly longer (56.0 ± 1.7 mm and 56.0 ± 1.8 mm, respectively), while the mean TL in normal years was significantly shorter (54.7 ± 1.8 mm) (Fig 4b).

### Long-term annual variation in the TL of glass eel

**Fig 5:**
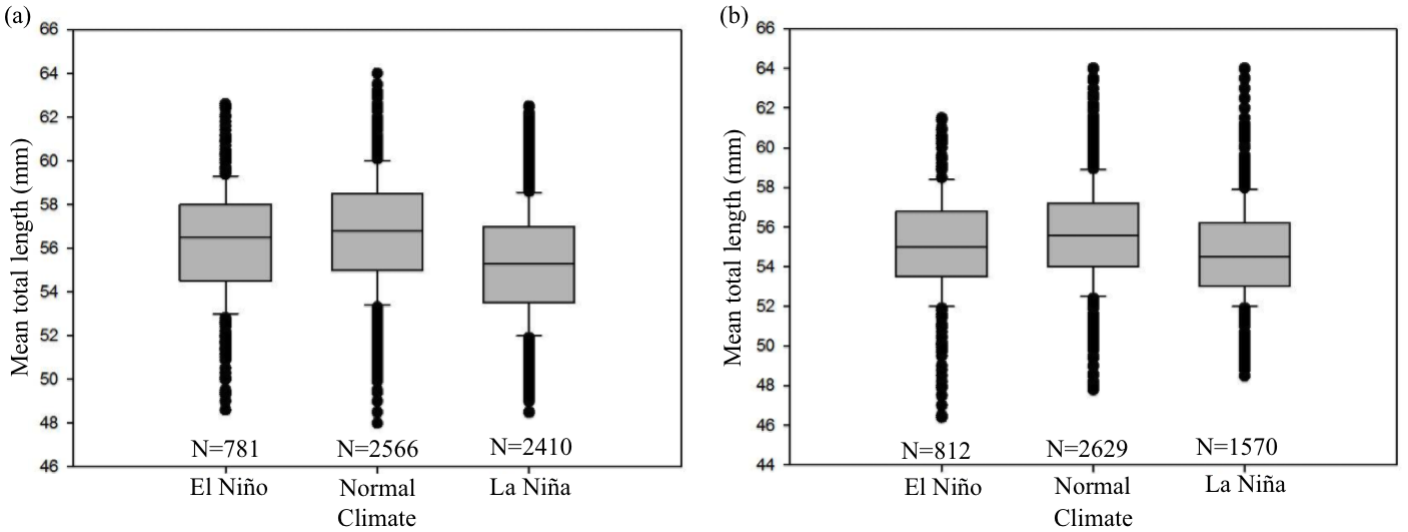
Comparison of the mean total length (after preservation shrinkage) between different ENSO climate periods for *A. japonica* glass eels collected in Taiwan from 1984 to 2013. (a) Stage *V*_*A*_ (N = 5757); (b) Stage *V*_*B*_ (N = 5011). Bar charts with different letters above are significantly different (Duncan, *p* < 0.05).

Because the mean TL between stage VA and VB glass eels collected in six Taiwanese estuaries in 30 years was significantly different [32], we statistically analyzed the specimens’ sizes separately for stages VA and VB eels. According to the statistical information of 30 years, the mean TL of the three climatic conditions (El Niño, La Niña, and normal) showed a significant difference in TL for both stage VA and stage VB eels (Duncan, *p* < 0.05; Fig 5a and 5b). In stage VA, the mean TL in normal years (56.7 ± 2.6 mm) was the longest, followed by that in El Niño years (56.3 ± 2.5 mm), and the shortest being that in in La Niña years (55.4 ± 2.5 mm); in stage VB, the mean TLs in the El Niño and normal years were significantly longer (55.6 ± 2.7 mm and 55.6 ± 2.6 mm, respectively), whereas that in the La Niña years was significant shorter (54.8 ± 2.4 mm) (Duncan, *p* < 0.05).

### Relationships between particle modeling and glass eel recruitment data

**Fig 6:**
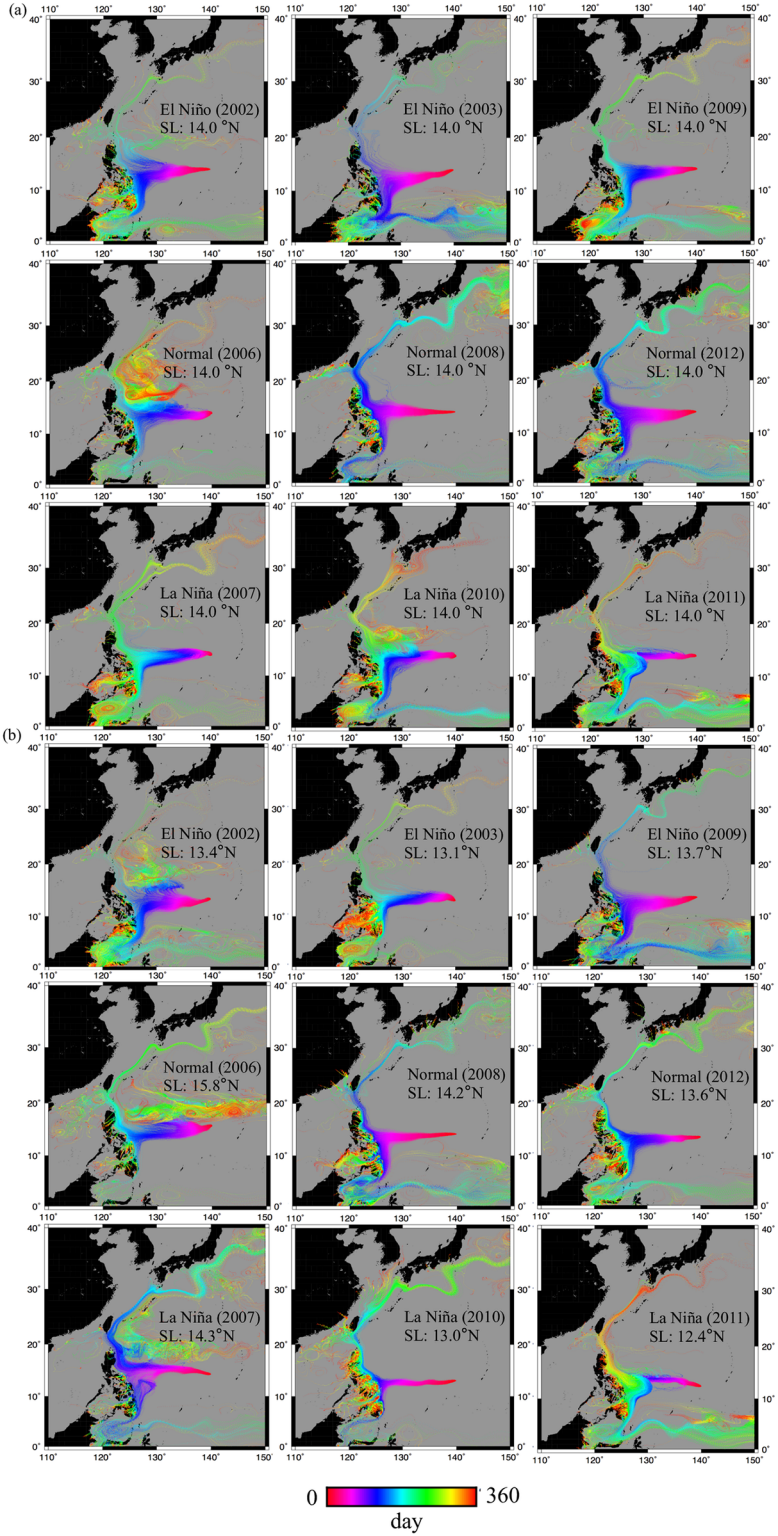
Particle trajectories released at the spawning ground with different ENSO climate years. Particles are released from (a) a fixed spawning ground of 14°N and (b) changing spawning grounds associated with latitude of the salinity front along 142°N. Spawning latitudes (SL) are the release points for simulation changed spawning grounds.

**Fig 7:**
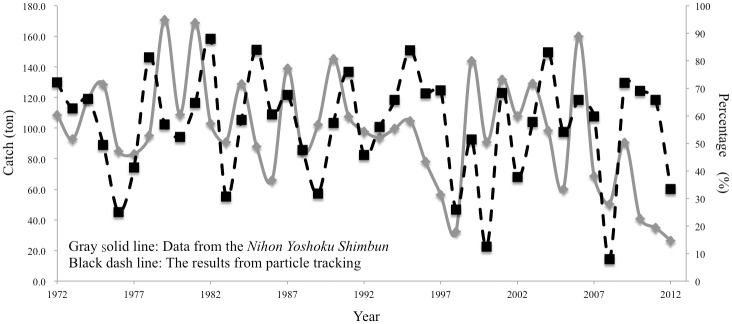
Comparison of annual commercial catches of glass eel (tonnes) from East Asian countries (grey solid line, data from the Nihon Yoshoku Shimbun) and the percentage of released particles in the transport modelling study that entered the Kuroshio Current (black dashed line).

Fig 6 shows particle trajectories released at the spawning ground over time for both for fixed on 14°N (Fig 6a) and vary with salinity front (Fig 6b). The percentage of particles released that entered the KC was significantly lower during the El Niño years, while the percentage of particles released that entered the MC was higher. Fig 7 shows that the two datasets exhibited roughly similar peaks and troughs, although they did not exactly correspond to one another.

### Numerical simulation of larval transport

**Fig 8:**
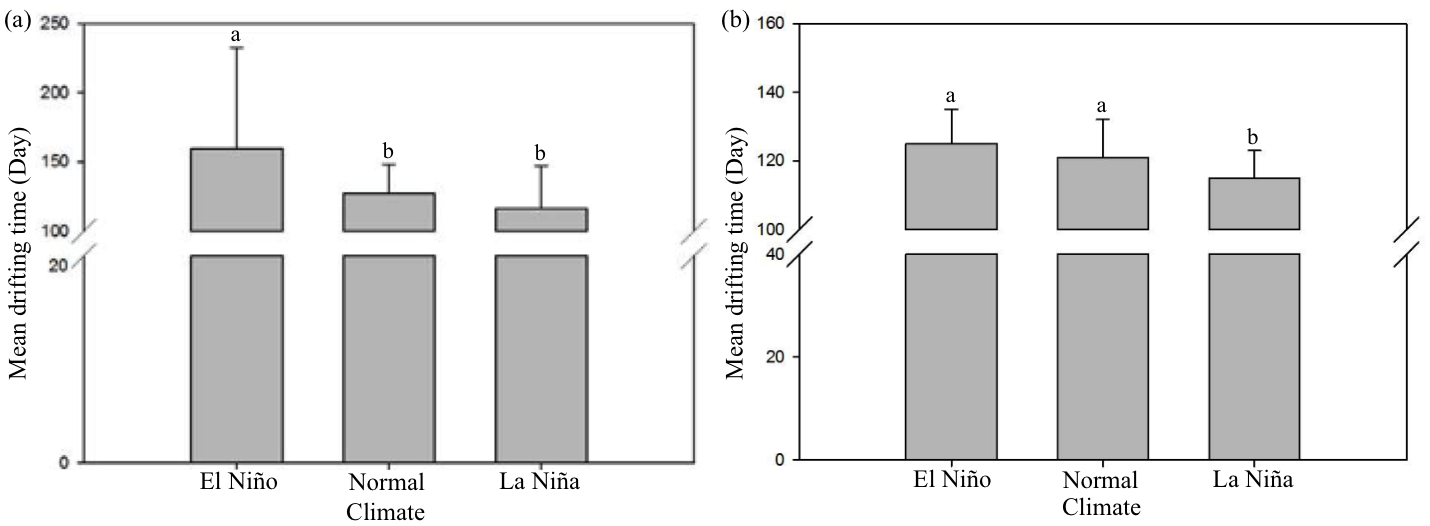
Comparison of the mean drifting time among El Niño, normal and La Niña years. Starting spawning area locations are: (a) a fixed spawning ground of 14°N and (b) at the latitude of the salinity front along the 142°E longitude.

Numerous previous studies have indicated that ENSO events influence oceanic and atmospheric conditions, especially relative to the capacity and velocity of the currents. Our results showed that particles released in the El Niño years from a fixed latitude of 14° N required the longest time (126.5 ± 34.6 days) for glass eel recruitment to Taiwanese estuaries, followed by normal (122.5 ± 24.2 days) and La Niña (115.3 ± 26.4 days) years (Fig 8a). However, when the spawning location of *A. japonica* was changed according to the position of the salinity front, particles released in the El Niño and normal years required the longest time for recruitment (125 ± 30.3 and 121 ± 33.6 days), and those in the La Niña years (115 ± 22.8 days) required the shortest time (Fig 8b).

**Fig 9:**
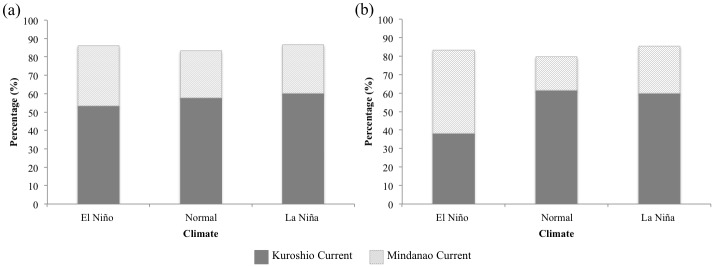
Comparison of the recruitment rate among El Niño, normal and La Niña years. Starting spawning area locations are: (a) a fixed spawning ground of 14°N and (b) changing spawning grounds associated with latitude of salinity front along 142°E into Kuroshio Current (dark grey solid bar) and Mindanao Current (light grey slashed bar).

Fig 9a and 9b show the percentage of particles transported into the KC and MC when released from the fixed latitude (14° N). The lowest percentage of particles was transported to the KC during the El Niño years whereas the highest was transported to the MC, but no significant difference was observed between normal and La Niña years (Fig 9a). However, when the spawning location changed with respect to the salinity front, the percentage of particles transported to the KC was significantly lower (38.3%) in the El Niño years, whereas it was significantly higher in normal and La Niña years (61.5% and 60%, respectively). In contrast, the percentage of particles transported to the MC was significantly highest in the El Niño years (45.1%), followed by La Niña (24.5%) and normal (18%) years (Fig 9b).

## Discussion

This study examined the effects of ENSO events on LD and transport, mainly using three methods: otolith analysis, TL analysis, and particle tracking via numerical modeling.

### The effects of ENSO events on the TL and LD of glass eel

The relationship between mean TL and LD in this study was represented by a linear regression. Our results were consistent with those of previous studies on the tropical eel, *A. marmorata*, which showed a positive correlation between TL (mm) and age (days) during early life stages [29].

Previous studies have mainly focused on the relationship between annual eel catch and ENSO events, but the influence of ENSO events on the TL and LD of the Japanese glass eel has seldom been reported. In this study, we show that LD in oceanic current systems is significantly longer during the El Niño years, implying that ENSO events affect the LD in this species. However, the TL and LD were not correlated when the sample size was small (N = 182). Although the TL of larvae was affected by ENSO events (indicated by our linear regression analysis), fluctuations in TL could be affected by many other factors, such as marine primary productivity, feeding status, and water temperature. In addition, differences in sampling locations and month may also cause fluctuations in TL. However, it is worth noting that the *A. japonica* specimens we analyzed were survivors that were collected from the estuaries. This means that slow-growing eels and those with long drifting time have been filtered out. This may be an explanation of why despite the statistically significant differences in TL and LD, their actual differences were not apparent. Therefore, it may be more difficult to explain the direct effects of El Niño events on the specimen size, especially when the sample size is not sufficiently large.

Our results showed that the TL of Japanese eel is proportional to their daily age during the leptocephali stage. According to the previous studies, although the growth of the body length was highly correlated with daily age, and demonstrates a linear relationship before metamorphosis [49–51], there are many environmental factors that can affect the body length in the environment. On the other hand, previous studies have critically reviewed how well otolith growth reflects fish age and growth at the daily level precision [25, 52]. Maillet and Checkle [53] further demonstrated that otoliths growth is maintained even through periods when somatic growth is nonexistent (i.e. no feeding, low water temperature or poor growth situation). Thus, the growth of the body length was not as accurate as that in the otolith increments, it might be harder to see significant differences while the sample size of TL is small. However, when we examined a larger sample size (comprising 10,768 specimens), we found differences in the mean TL of glass eels among the El Niño, La Niña, and normal years, suggesting that ENSO events have likely affected the specimen size. Although the highest mean TL in stage VA specimens was observed in normal climate years, the mean TL in the La Niña years was significantly lower in both stages VA and VB. Therefore, the El Niño and La Niña events have positive and negative influences on the LD and TL of Japanese glass eel, respectively. There are a number of explanations for this result: The spawning ground of *A. japonica* is located to the south of the salinity front; thus, the location of the salinity front may be crucial to the success of their spawning migration [18]. Kimura et al. [16] showed certain synchrony between *A. japonica* recruitment and salinity front driven by ENSO events in the Japanese eel spawning area. If the spawning area moves southward associated with movement of the salinity front, leptocephali have to be transported by the southern part of NEC and larvae are more likely to enter the Mindanao region [18]. During the El Niño event of 2002, Kimura and Tsukamoto [18] noticed that small larvae (*TL* < 10 mm) were collected just south of the salinity front, where these larvae had never been found during normal climatic years. Taken together, the interannual variability of the salinity front associated with ENSO events probably leads to the reduced larval transport into the KC, causing poor recruitment into estuaries in eastern Asia [16].

### The effects of ENSO events on NEC and NEC bifurcation

The latitude of NEC bifurcation provides an indicator of how NEC mass, heat, and salinity transport are partitioned between the KC and MC. Furthermore, Kim et al. [17] suggested that the meridional migration of NEC bifurcation is strongly influenced by ENSO events. When NEC bifurcation moves northward during the El Niño years, more NEC water flows into the MC. Conversely, a large amount of water is transported to the Kuroshio region during the La Niña years when NEC bifurcation shifts southward. Kimura et al. [54] also suggested that the meridional variation in NEC bifurcation could influence the recruitment of Japanese eels into the East China Sea.

### Relationship between particle tracking and glass eel recruitment data

There are many factors that could be considered to cause effects on Japanese eel recruitment dynamics, including the following: timing of the metamorphosis of leptocephali, the KC and NEC velocity, effects of eddies, movement of the NEC bifurcation and salinity front, oceanic primary production, sea-surface temperature, depth of mixed layer, habitat destruction, overfishing, and interannual climate events (i.e. ENSO events, Pacific Decadal Oscillation, PDO and Philippines-Taiwan Oscillation, PTO) [3, 20, 24, 55–60]. Glass eel recruitment data for East Asia showed fluctuation in peaks and troughs that partly mirror those for the percentage of particles entering the KC in our particle tracking simulation (Fig 7).

### Effects of ENSO events on the numerical simulation of larval transport

The results of the mean drifting time and transport success rate showed that it took the longest time for eels to get transported to the KC from both the fixed and variable spawning ground. In addition, the KC entrance success rate was the lowest during the El Niño years, when the releasing latitude changed with the salinity front. These results also correspond to the previous study that used a circulation field model called the high-resolution ocean model for the Earth Simulator (OFES), based on the Modular Ocean Model (MOM3) and developed by the Geophysical Fluid Dynamics Laboratory (GFDL) of NOAA to simulate the particle transport from the spawning ground of *A. japonica* to the KC [24]. Detailed descriptions of the basic settings of the model are given by Masumoto et al. [61], Kim et al. [17] and Sasaki et al. [60]. The results revealed that particle transport to the KC was the lowest during the two El Niño years among all years studied [24]. Additionally, particle transport to the KC decreased when the particles were released at the variable salinity front in an El Niño year. The correspondence of these simulation results between previous and current studies using different circulation models further demonstrated their reliability. Another study also showed that larval transport in the El Niño year decreased to half of that in a normal and La Niña years, when the movement of salinity front changed the location of the spawning ground [17].

In a recent study, Hsu et al. [59] demonstrated that although the yearly-averaged KC entrance success rate, determined through particle tracking experiments, does not correspond with ENSO events, these events affect the NEC bifurcation location and velocity of the NEC. Unlike the Model for Interdisciplinary Research on Climate (MIROC), the Regional Ocean Modeling System (ROMS), used by Hsu et al. [59], has been primarily utilized in regional and mesoscale studies. The Japanese eel (*Anguilla japonica*) is distributed in the western Pacific Ocean and migrates about 3000 km away from their spawning ground to their habitats. Therefore, the MIROC used in the current study might be more suitable than the ROMS for particle tracking experiments of Japanese eels. Furthermore, analyses of otolith increment and body length indicated that the obvious differences observed in these parameters were related to ENSO events. As a result, the combined effects of the salinity front and NEC bifurcation on the recruitment of *A. japonica* glass eels were quite apparent. As claimed by previous studies, the shifting of NEC bifurcation latitude clearly correlates with ENSO events. This suggests that the duration of recruitment of *A. japonica* might have been affected by the alteration of the bifurcation and salinity front latitude. However, Wu [62] demonstrated that ENSO events might not be entirely responsible for the atmospheric variability over the north-western Pacific. Other factors that affect the recruitment of eels are related to the changes in oceanic conditions.

### Other climate events that might affect glass eel recruitment dynamics

According to the previous study, Qiu and Chen [63] analyzed long-term time series of the NEC bifurcation latitudes. They noted that the Niño-3.4 index explains only around 25% of the variance in latitudes of the NEC bifurcation. This implies that a climate phenomenon other than ENSO events might also play a role in modulating the meridional migration of the NEC bifurcation latitude. Mantua et al. [64] further noted that the interannual relationship between ENSO events and global climate is not stationary and can be modulated by the Pacific Decadal Oscillation (PDO). The interannual relationship between ENSO events and winter monsoons is weak and insignificant in the warm phase of the PDO. The time-varying NEC bifurcation latitude is also in agreement with the concurrent PDO indices. However, an ENSO event exerts a strong impact on the winter monsoon in the cold phase of the PDO, when the variability in the NEC bifurcation latitude is best represented by the Niño-3.4 index [62]. In a recent study, Chang et al. [65] demonstrated that the recruitment index of the arrival of Japanese eel in Taiwanese estuaries is higher in the negative Philippines-Taiwan Oscillation (PTO) years (1996, 2002, and 2003), when the NEC moved north and overlapped with the spawning ground, and lower in the positive PTO years (1998, 2000, and 2007). Hsu et al. [59] further reported that the yearly average percentage of particles entering the KC is controlled by the average zonal velocity of a fixed domain (125–143°E, 13.5–17°N), which is also highly correlated with PTO in the 20-year study period (1993-2012).

## Conclusion

This study builds upon previous insights on the effects of ENSO events on the recruitment dynamics, time, and body size of Japanese eels by analyzing long-term otolith samples and performing particle tracking experiment using high-resolution MIROC data. Future research should analyze eel recruitment over a longer time frame and consider the interactive effects between a wider variety of climatic events. Circulation models with higher resolution and experimental design for particle tracking that clearly reflect the actual situation could further improve the predictions of fluctuations in Japanese eel recruitment dynamics, thereby contributing to eel management, policy formulation, and related research.

## References

[1] Tesch FW, White RJ. The eel. John Wiley & Sons; 2008.

[2] Kuroki M, Aoyama J, Miller M, Yoshinaga T, Shinoda A, Hagihara S, et al Sympatric spawning of Anguilla marmorata and Anguilla japonica in the western North Pacific Ocean. Journal of Fish Biology. 2009;74(9):1853–1865. doi: 10.1111/j.1095-8649.2009.02299.x 20735676 10.1111/j.1095-8649.2009.02299.x

[3] Cheng P, Tzeng W. Timing of metamorphosis and estuarine arrival across the dispersal range of the Japanese eel Anguilla japonica. Marine Ecology Progress Series. 1996; p. 87–96. doi: 10.3354/meps131087

[4] Tsukamoto K. Discovery of the spawning area for Japanese eel. Nature. 1992;356(6372):789–791. doi: 10.1038/356789a0

[5] Tsukamoto K. Otolith daily increments in the Japanese eel. Bull Jpn Soc Sci Fish. 1989;55:1017–1021. doi: 10.2331/suisan.55.1017

[6] Han YS, Zhang H, Tseng YH, Shen ML. Larval Japanese eel (Anguilla japonica) as sub-surface current bio-tracers on the East Asia continental shelf. Fisheries Oceanography. 2012;21(4):281–290. doi: 10.1111/j.1365-2419.2012.00624.x

[7] Tsukamoto K. Oceanic biology: spawning of eels near a seamount. Nature. 2006;439(7079):929 doi: 10.1038/439929a 16495988 10.1038/439929a

[8] McCleave JD, Wippelhauser GS. Behavioral aspects of selective tidal stream transport in juvenile American eels. In: American Fisheries Society Symposium. vol. 1; 1987. p. 138–150.

[9] Miller MJ, Kimura S, Friedland KD, Knights B, Kim H, Jellyman DJ, et al. Review of ocean-atmospheric factors in the Atlantic and Pacific oceans influencing spawning and recruitment of anguillid eels. In: Challenges for Diadromous Fishes in a Dynamic Global Environment. American Fisheries Society Symposium. vol. 69; 2009. p. 231–249.

[10] Tatsukawa K. Eel resources in east Asia In: Eel biology. Springer; 2003 p. 293–298.

[11] Tzeng WN, Tseng YH, Han YS, Hsu CC, Chang CW, Di Lorenzo E, et al Evaluation of multi-scale climate effects on annual recruitment levels of the Japanese eel, Anguilla japonica, to Taiwan. Plos One. 2012;7(2):e30805 doi: 10.1371/journal.pone.0030805 22383976 10.1371/journal.pone.0030805 PMC3285622

[12] Jacoby D, Gollock M. Anguilla anguilla. The IUCN red list of threatened species. Version 2014.2; 2014.

[13] Chen JZ, Huang SL, Han YS. Impact of long-term habitat loss on the Japanese eel Anguilla japonica. Estuarine, Coastal and Shelf Science. 2014;151:361–369. doi: 10.1016/j.ecss.2014.06.004

[14] Itakura H, Kaino T, Miyake Y, Kitagawa T, Kimura S. Feeding, condition, and abundance of Japanese eels from natural and revetment habitats in the Tone River, Japan. Environmental biology of fishes. 2015;98(8):1871–1888. doi: 10.1007/s10641-015-0404-6

[15] Itakura H, Kitagawa T, Miller MJ, Kimura S. Declines in catches of Japanese eels in rivers and lakes across Japan: Have river and lake modifications reduced fishery catches? Landscape and ecological engineering. 2015;11(1):147–160. doi: 10.1007/s11355-014-0252-0

[16] Kimura S, Inoue T, Sugimoto T. Fluctuation in the distribution of low-salinity water in the North Equatorial Current and its effect on the larval transport of the Japanese eel. Fisheries Oceanography. 2001;10(1):51–60. doi: 10.1046/j.1365-2419.2001.00159.x

[17] Kim H, Kimura S, Shinoda A, Kitagawa T, Sasai Y, Sasaki H. Effect of El Niño on migration and larval transport of the Japanese eel (Anguilla japonica). ICES Journal of Marine Science. 2007;64(7):1387–1395. doi: 10.1093/icesjms/fsm091

[18] Kimura S, Tsukamoto K. The salinity front in the North Equatorial Current: a landmark for the spawning migration of the Japanese eel (Anguilla japonica) related to the stock recruitment. Deep Sea Research Part II: Topical Studies in Oceanography. 2006;53(3-4):315–325. doi: 10.1016/j.dsr2.2006.01.009

[19] Kimura S, Döös K, Coward AC. Numerical simulation to resolve the issue of downstream migration of the Japanese eel. Marine Ecology Progress Series. 1999;186:303–306. doi: 10.3354/meps186303

[20] Miller MJ, Tsukamoto K. The ecology of oceanic dispersal and survival of anguillid leptocephali. Canadian Journal of Fisheries and Aquatic Sciences. 2016;74(6):958–971. doi: 10.1139/cjfas-2016-0281

[21] Nitani H. Beginning of the Kuroshio Kuroshio, Physical Aspect of the Japan Current. 1972;.

[22] Toole JM, Millard RC, Wang Z, Pu S. Observations of the Pacific North Equatorial Current bifurcation at the Philippine coast. Journal of Physical Oceanography. 1990;20(2):307–318. doi: 10.1175/1520-0485(1990)020%3C0307:OOTPNE%3E2.0.CO;2

[23] Kim YY, Qu T, Jensen T, Miyama T, Mitsudera H, Kang HW, et al Seasonal and interannual variations of the North Equatorial Current bifurcation in a high-resolution OGCM. Journal of Geophysical Research: Oceans. 2004;109(C3). doi: 10.1029/2003JC002013

[24] Zenimoto K, Kitagawa T, Miyazaki S, Sasai Y, Sasaki H, Kimura S. The effects of seasonal and interannual variability of oceanic structure in the western Pacific North Equatorial Current on larval transport of the Japanese eel Anguilla japonica. Journal of Fish Biology. 2009;74(9):1878–1890. doi: 10.1111/j.1095-8649.2009.02295.x 20735678 10.1111/j.1095-8649.2009.02295.x

[25] Pannella G. Fish otoliths: daily growth layers and periodical patterns. Science. 1971;173(4002):1124–1127. doi: 10.1126/science.173.4002.1124 5098955 10.1126/science.173.4002.1124

[26] Campana SE. Feeding periodicity and the production of daily growth increments in otoliths of steelhead trout (Salmo gairdneri) and starry flounder (Platichthys stellatus). Canadian Journal of Zoology. 1983;61(7):1591–1597. doi: 10.1139/z83-214

[27] Townsend CR, Hildrew AG. Foraging in a patchy environment by a predatory net-spinning caddis larva: a test of optimal foraging theory. Oecologia. 1980;47(2):219–221. doi: 10.1007/BF00346824 28309475 10.1007/BF00346824

[28] Umezawa A, Tsukamoto K, Tabeta O, Yamakawa H. Daily growth increments in the larval otolith of the Japanese eel, Anguilla japonica. Japanese Journal of Ichthyology. 1989;35(4):440–444.

[29] Kuroki M, Aoyama J, Miller MJ, Arai T, Sugeha HY, Minagawa G, et al Correspondence between otolith microstructural changes and early life history events in Anguilla marmorata leptocephali and glass eels. Mar Sci. 2005;29:154–161.

[30] Ando K, McPhaden MJ. Variability of surface layer hydrography in the tropical Pacific Ocean. Journal of Geophysical Research: Oceans. 1997;102(C10):23063–23078. doi: 10.1029/97JC01443

[31] Kessler WS, Taft BA. Dynamic heights and zonal geostrophic transports in the central tropical Pacific during 1979–84. Journal of Physical Oceanography. 1987;17(1):97–122. doi: 10.1175/1520-0485(1987)017%3C0097:DHAZGT%3E2.0.CO;2

[32] Ho M. Spatial and temporal variability in length of Japanese glass eel, Anguilla japonica; Master dissertation, National Taiwan University. 2012. Available from: http://handle.ncl.edu.tw/11296/ndltd/38032923852727127189.

[33] Cheng P, Tzeng WN. Timing of metamorphosis and estuarine arrival across the dispersal range of the Japanese eel Anguilla japonica. Marine Ecology Progress Series. 1996; p. 87–96. doi: 10.3354/meps131087

[34] Han YS, Tzeng WN, Liao IC, et al Time series analysis of Taiwanese catch data of Japanese glass eels Anguilla japonica: possible effects of the reproductive cycle and El Niño events. Zool Stud. 2009;48(5):632–639.

[35] Tzeng WN. Immigration timing and activity rhythms of the eel, Anguilla japonica, elvers in the estuary of northern Taiwan, with emphasis on environmental influences. Bull Jpn Soc Fish Oceanogr. 1985;47(48):11–28.

[36] Tzeng WN. Relationship between growth rate and age at recruitment ofAnguilla japonica elvers in a Taiwan estuary as inferred from otolith growth increments. Marine Biology. 1990;107(1):75–81. doi: 10.1007/BF01313244

[37] K-1 model developers. K-1 coupled gcm (miroc) description; KI Technical Report. 2004;1. Available from: http://ccsr.aori.u-tokyo.ac.jp/~hasumi/miroc_description.pdf.

[38] Kawamiya M, Yoshikawa C, Kato T, Sato H, Sudo K, Watanabe S, et al Development of an integrated Earth system model on the Earth Simulator. J Earth Simulator. 2005;4:18–30.

[39] Nakicenovic N, Alcamo J, Grubler A, Riahi K, Roehrl R, Rogner HH, et al. Special report on emissions scenarios (SRES), a special report of Working Group III of the intergovernmental panel on climate change. Cambridge University Press; 2000.

[40] Sakamoto TT, Komuro Y, Nishimura T, Ishii M, Tatebe H, Shiogama H, et al MIROC4h—a new high-resolution atmosphere-ocean coupled general circulation model. Journal of the Meteorological Society of Japan Ser II. 2012;90(3):325–359. doi: 10.2151/jmsj.2012-301

[41] Hasumi H. CCSR ocean component model (COCO). CCSR Rep. 2000;13:68.

[42] Tatebe H, Ishii M, Mochizuki T, Chikamoto Y, Sakamoto TT, Komuro Y, et al The initialization of the MIROC climate models with hydrographic data assimilation for decadal prediction. Journal of the Meteorological Society of Japan Ser II. 2012;90:275–294. doi: 10.2151/jmsj.2012-A14

[43] Tsukamoto K. Recruitment mechanism of the eel, Anguilla japonica, to the Japanese coast. Journal of Fish Biology. 1990;36(5):659–671. doi: 10.1111/j.1095-8649.1990.tb04320.x

[44] Osame T, Kuniaki T, Juro Y, Wann-Nian T. Aspects of the early life history of the Japanese eel Anguilla japonica determined from otolith microstructure. NIPPON SUISAN GAKKAISHI. 1987;53(10):1727–1734. doi: 10.2331/suisan.53.1727

[45] McCleave JD. Contrasts between spawning times of Anguilla species estimated from larval sampling at sea and from otolith analysis of recruiting glass eels. Marine Biology. 2008;155(3):249 doi: 10.1007/s00227-008-1026-8

[46] Castonguay M, McCleave JD. Vertical distributions, diel and ontogenetic vertical migrations and net avoidance of leptocephali of Anguilla and other common species in the Sargasso Sea. Journal of Plankton Research. 1987;9(1):195–214. doi: 10.1093/plankt/9.1.195

[47] Kajihara T. Distribution of Anguilla japonica leptocephali in western Pacific during September 1986. Nippon Suisan Gakkaishi. 1988;54(6):929–933. doi: 10.2331/suisan.54.929

[48] Otake T. Metamorphosis In: Eel biology. Springer; 2003 p. 61–74.

[49] Tsukamoto K, Umezawa A, Ozawa T. Age and Growth of Anguilla japonica Leptocephali Collected in Western North Pacific in July 1990. Nippon Suisan Gakkaishi. 1992;58(3):457–459. doi: 10.2331/suisan.58.457

[50] Ishikawa S, Suzuki K, Inagaki T, Watanabe S, Kimura Y, Okamura A, et al Spawning time and place of the Japanese eel Anguilla japonica in the North Equatorial Current of the western North Pacific Ocean. Fisheries science. 2001;67(6):1097–1103. doi: 10.1046/j.1444-2906.2001.00366.x

[51] Shinoda A, Tanaka H, Kagawa H, Ohta H, Tsukamoto K. Otolith microstructural analysis of reared larvae of the Japanese eel Anguilla japonica. Fisheries science. 2004;70(2):339–341. doi: 10.1111/j.1444-2906.2003.00810.x

[52] Campana SE, Neilson JD. Microstructure of fish otoliths. Canadian Journal of Fisheries and Aquatic Sciences. 1985;42(5):1014–1032. doi: 10.1139/f85-127

[53] Maillet G. Effects of starvation on the frequency of formation and width of growth increments in sagittae of laboratory-reared Atlantic menhaden Brevoortia tyrannus larvae. Fish Bull. 1989;88:155–165.

[54] Kimura S, Tsukamoto K, Sugimoto T. A model for the larval migration of the Japanese eel: roles of the trade winds and salinity front. Marine Biology. 1994;119(2):185–190. doi: 10.1007/BF00349555

[55] Knights B. A review of the possible impacts of long-term oceanic and climate changes and fishing mortality on recruitment of anguillid eels of the Northern Hemisphere. Science of the total Environment. 2003;310(1-3):237–244. doi: 10.1016/S0048-9697(02)00644-7 12812748 10.1016/S0048-9697(02)00644-7

[56] Friedland KD, Miller MJ, Knights B. Oceanic changes in the Sargasso Sea and declines in recruitment of the European eel. ICES Journal of Marine Science. 2007;64(3):519–530. doi: 10.1093/icesjms/fsm022

[57] Bonhommeau S, Chassot E, Planque B, Rivot E, Knap AH, Le Pape O. Impact of climate on eel populations of the Northern Hemisphere. Marine Ecology Progress Series. 2008;373:71–80. doi: 10.3354/meps07696

[58] Kettle AJ, Bakker DC, Haines K. Impact of the North Atlantic Oscillation on the trans-Atlantic migrations of the European eel (Anguilla anguilla). Journal of Geophysical Research: Biogeosciences. 2008;113(G3). doi: 10.1029/2007JG000589

[59] Hsu AC, Xue H, Chai F, Xiu P, Han YS. Variability of the Pacific North Equatorial Current and its implications on Japanese eel (Anguilla japonica) larval migration. Fisheries Oceanography. 2017;26(3):251–267. doi: 10.1111/fog.12189

[60] Sasaki H, Sasai Y, Nonaka M, Masumoto Y, Kawahara S. An eddy-resolving simulation of the quasi-global ocean driven by satellite-observed wind field. Journal of the Earth Simulator. 2006;6:35–49.

[61] Masumoto Y, Sasaki H, Kagimoto T, Komori N, Ishida A, Sasai Y, et al A fifty-year eddy-resolving simulation of the world ocean: Preliminary outcomes of OFES (OGCM for the Earth Simulator). J Earth Simulator. 2004;1:35–56.

[62] Wu CR. Interannual modulation of the Pacific Decadal Oscillation (PDO) on the low-latitude western North Pacific. Progress in Oceanography. 2013;110:49–58. doi: 10.1016/j.pocean.2012.12.001

[63] Qiu B, Chen S. Interannual-to-decadal variability in the bifurcation of the North Equatorial Current off the Philippines. Journal of Physical Oceanography. 2010;40(11):2525–2538. doi: 10.1175/2010JPO4462.1

[64] Mantua NJ, Hare SR, Zhang Y, Wallace JM, Francis RC. A Pacific interdecadal climate oscillation with impacts on salmon production. Bulletin of the american Meteorological Society. 1997;78(6):1069–1079. doi: 10.1175/1520-0477(1997)078%3C1069:APICOW%3E2.0.CO;2

[65] Chang YL, Sheng J, Ohashi K, Béguer-Pon M, Miyazawa Y. Impacts of interannual ocean circulation variability on Japanese eel larval migration in the western North Pacific Ocean. PloS one. 2015;10(12):e0144423 doi: 10.1371/journal.pone.0144423 26642318 10.1371/journal.pone.0144423 PMC4671650

[66] Arai T, Otake T, Tsukamoto K. Timing of metamorphosis and larval segregation of the Atlantic eels Anguilla rostrata and A. anguilla, as revealed by otolith microstructure and microchemistry. Marine Biology. 2000;137(1):39–45. doi: 10.1007/s002270000326

